# Machine learning models identify molecules active against the Ebola virus *in vitro*

**DOI:** 10.12688/f1000research.7217.3

**Published:** 2017-01-17

**Authors:** Sean Ekins, Joel S. Freundlich, Alex M. Clark, Manu Anantpadma, Robert A. Davey, Peter Madrid

**Affiliations:** 1 Collaborations in Chemistry, Fuquay-Varina, NC, 27526, USA; 2 Collaborations Pharmaceuticals Inc, Fuquay-Varina, NC, 27526, USA; 3 Collaborative Drug Discovery, Burlingame, CA, 94010, USA; 4 Departments of Pharmacology & Physiology and Medicine, Center for Emerging and Reemerging Pathogens, UMDNJ, New Jersey Medical School, Newark, NJ, 07103, USA; 5 Molecular Materials Informatics, Inc., Montreal, 94025, Canada; 6 Texas Biomedical Research Institute, San Antonio, TX, 78227, USA; 7 SRI International, Menlo Park, CA, 94025, USA

**Keywords:** Drug repurposing, Ebola Virus, Computational models, Machine learning, Pharmacophore, Pyronaridine, Quinacrine, Tilorone

## Abstract

The search for small molecule inhibitors of Ebola virus (EBOV) has led to several high throughput screens over the past 3 years. These have identified a range of FDA-approved active pharmaceutical ingredients (APIs) with anti-EBOV activity *in vitro* and several of which are also active in a mouse infection model. There are millions of additional commercially-available molecules that could be screened for potential activities as anti-EBOV compounds. One way to prioritize compounds for testing is to generate computational models based on the high throughput screening data and then virtually screen compound libraries. In the current study, we have generated Bayesian machine learning models with viral pseudotype entry assay and the EBOV replication assay data. We have validated the models internally and externally. We have also used these models to computationally score the MicroSource library of drugs to select those likely to be potential inhibitors. Three of the highest scoring molecules that were not in the model training sets, quinacrine, pyronaridine and tilorone, were tested *in vitro* and had EC _50_ values of 350, 420 and 230 nM, respectively. Pyronaridine is a component of a combination therapy for malaria that was recently approved by the European Medicines Agency, which may make it more readily accessible for clinical testing. Like other known antimalarial drugs active against EBOV, it shares the 4-aminoquinoline scaffold. Tilorone, is an investigational antiviral agent that has shown a broad array of biological activities including cell growth inhibition in cancer cells, antifibrotic properties, α7 nicotinic receptor agonist activity, radioprotective activity and activation of hypoxia inducible factor-1. Quinacrine is an antimalarial but also has use as an anthelmintic. Our results suggest data sets with less than 1,000 molecules can produce validated machine learning models that can in turn be utilized to identify novel EBOV inhibitors *in vitro*.

## Introduction

In 2014, the outbreak of the Ebola virus (EBOV) in West Africa highlighted the need for broad-spectrum antiviral drugs for this and other emerging viruses ^1^. Several groups had previously performed high throughput screens (HTS) and identified FDA approved drugs (amodiaquine, chloroquine, clomiphene and toremifene) with *in vitro* growth inhibitory activities against EBOV ^2, 3^. It appears none of these molecules were tried during the epidemic in Africa ^4^, likely due to the lack of efficacy data in higher order species. We have previously summarized the numerous small molecules described in the literature as possessing antiviral activity that could be further evaluated for their potential EBOV activity alongside the few new antivirals. We have found that there is considerable prior knowledge regarding these small molecules possessing activity against EBOV *in vitro* or in animal models ^5– 8^, and this includes a number of accessible FDA-approved drugs ^2, 3, 9^. Another recent study has shown three approved ion channel blockers (amiodarone, dronedarone, and verapamil) inhibited EBOV cellular entry ^9^. The drugs were given at concentrations that would be achieved in human serum, and were effective against several of the filoviruses ^9^. None of the FDA approved drugs described in these various studies were designed to target the Ebola virus. For example amodiaquine and chloroquine are well known antimalarials, clomiphene and toremifene are selective estrogen receptor modulators, while amiodarone, dronedarone, and verapamil are anti-arrhythmics ^4^. It may or may not be of importance but all of these compounds have a common tertiary amine feature ^10, 11^. What is important is that they are all orally bioavailable and generally safe for humans at their approved doses. Some have suggested that G-protein-coupled receptors (GPCRs) may play a role in filoviral entry and receptor antagonists could be developed as anti-EBOV therapies ^12^. The compounds which are FDA-approved drugs for other diseases ^2, 3, 9^ but with activity against EBOV *in vitro* or *in vivo* may represent useful starting points with the advantage that much is known regarding their absorption, distribution, metabolism and excretion (ADME) and toxicity properties. Thus, these repurposed drugs may represent a more advanced starting point for therapeutic development and approval compared with new chemical entities for preventing the spread and mortality associated with EBOV.

Beyond these early stage drugs, there are a number of other compounds that have also been identified as active against EBOV (summarized in a review ^13^). A thorough literature search identified 55 molecules suggested to have activity against EBOV *in vitro* and/or *in vivo* which were evaluated from the perspective of an experienced medicinal chemist as well as using simple molecular properties and ultimately 16 were highlighted as desirable ^14^. This dataset overlaps to some extent with another review that identified over 60 molecules ^15^. Two recent repurposing screens identified 53 ^16^ and 80 ^17^ compounds with antiviral activity which also overlap the earlier screens. Additional studies have identified small number of inhibitors ^18, 19^. In total there may now be close to several hundred compounds identified with activity against EBOV *in vitro*.

Approaches with more capacity to screen compounds include using computational methods as a filter before *in vitro* testing. Computational models for anti-EBOV activity include one which used the average quasi valence number (AQVN) and the electron-ion interaction potential (EIIP), parameters determining long-range interaction between biological molecules for virtual screening of DrugBank and suggested hundreds of compounds to test ^20^. A follow up to this study proposed ibuprofen for testing ^21^. Others have also used computational docking studies to propose multi-target inhibitors of VP40, VP35, VP30 and VP24 ^22^, inhibitors of VP40 ^23^ or have suggested molecules to test in the absence of computational approaches ^24, 25^. We are unaware of any validation of these compounds. A further computational approach used a pharmacophore ^26^ that was generated from four FDA approved compounds resulting from the two earliest high throughput screens against EBOV ^2, 3^. This pharmacophore closely matched the receptor-ligand pharmacophores for the EBOV protein 35 (VP35) ^5^. Follow-up docking studies suggested that these compounds may also have favorable inhibitory interactions with this receptor. The pharmacophore was further used to screen several compound libraries ^27^. We proposed that if we could learn from the many compounds already screened for anti-EBOV activity, we could more efficiently find additional compounds and perhaps understand the key molecular features needed for antiviral activity ^14^. We speculated then that Laplacian-corrected Naïve Bayesian classifier models might be useful as they have been for *M. tuberculosis* ^28, 29^ and more recently for *T. cruzi* ^30^. To our knowledge machine learning approaches to identify EBOV inhibitors have not been attempted elsewhere. The current study extends the machine learning approach to EBOV and uses both commercially available Bayesian, Support Vector Machines (SVM) and recursive partitioning methods and open source Bayesian software for model generation and compound scoring. We report the identification of three novel EBOV inhibitors with nanomolar EC _50_ values as validation of this approach.

## Methods

### Chemicals and materials

Quinacrine hydrochloride, pyronaridine tetraphosphate, and tilorone dihydrochloride (BOC Sciences, Shirley, NY), bafilomycin A1, and chloroquine diphosphate (Sigma, St. Louis, MO) were dissolved in either DMSO or water as 10 mM stock solutions and were stored at -20°C. The nucleus staining dye, Hoechst 33342, CellMask Deep™ Red cytoplasmic/nuclear stain, NHS-Alexa-488 dye, the Dual-Glo® Luciferase Assay System and CytoTox 96™ assay kit were purchased from Promega (Promega, Madison, WI). The modified MTT assay Cell Counting Kit 8 was procured from Dojindo Molecular Technologies (Dojindo Molecular Technologies, Gaithersburg, MD). The 96-well high-content imaging plates were obtained from BD (BD Biosciences, Franklin Lakes, NJ) and 96-well white-walled tissue culture plates were from Corning (Corning Life Sciences, MA). The Opera QEHS confocal imaging reader, Acapella™ and Definiens™ image analysis packages were purchased from PerkinElmer (PerkinElmer, USA). Image acquisition was done using Nikon TI eclipse high content imaging enabled microscope running NIS elements high content imaging software (version 4.30.02).

### Machine learning

868 molecules from the viral pseudotype entry assay and the EBOV replication assay from a recent publication ^3, 31^ were made available as an sdf file ^3^. Salts were stripped and duplicates removed using Discovery Studio 4.1 (Biovia, San Diego, CA) ^32– 36^. For each assay, compounds with IC _50_ values less than 50 μM were selected as actives. All other compounds were classed as inactives. Models were generated using a standard protocol with the following molecular descriptors: molecular function class fingerprints of maximum diameter 6 (FCFP_6) ^37^, AlogP ^37a^, molecular weight, number of rotatable bonds, number of rings, number of aromatic rings, number of hydrogen bond acceptors, number of hydrogen bond donors, and molecular fractional polar surface area. Models were validated using five-fold cross validation (leave out 20% of the database). Bayesian, Support Vector Machine and Recursive Partitioning Forest and single tree models built with the same molecular descriptors in Discovery Studio were compared. For SVM models, we calculated interpretable descriptors in Discovery Studio and then used Pipeline Pilot to generate the FCFP_6 descriptors followed by integration with R ^38^. RP Forest and RP Single Tree models used the standard protocol in Discovery Studio. In the case of RP Forest models, ten trees were created with bagging. Bagging is short for “Bootstrap AGgregation”. For each tree, a bootstrap sample of the original data is taken, and this sample is used to grow the tree. RP Single Trees had a minimum of ten samples per node and a maximum tree depth of 20. In all cases, 5-fold cross validation or leave out 50% × 100 fold cross validation was used to calculate the Receiver Operator Curve (ROC) for the models generated ^28, 29^.

### Open Bayesian models

Open Bayesian models for the Ebola datasets were developed using open source software ^39– 41^ and loaded into the Mobile Molecular Data Sheet (MMDS ( http://molmatinf.com/)) and then the two models were used to score the three compounds selected by the earlier models. These two models are also openly accessible ( http://molsync.com/ebola/) and can be uploaded into MMDS in order to score molecules of interest.

### Pharmacophore mapping

Pyronaridine was mapped to the recently published pharmacophore ^26^ derived from Ebola *in vitro* inhibitors amodiaquine, chloroquine, clomiphene and toremifene in Discovery Studio Vers 4.1 and a fit score was generated.

### *In vitro* testing

Recombinant, infectious Ebola virus encoding green fluorescent protein (GFP) was used for testing efficacy of compounds and was originally provided by Dr. Heinz Feldmann, Rocky Mountain Laboratories. The strain that was used has the GFP gene inserted between the VP30 and VP24 genes. All viral infections were done in the BSL-4 lab at Texas Biomedical Research Institute. Briefly, 4,000 HeLa cells per well were grown overnight in 384-well tissue culture plates, the volume of DMEM (Fisher scientific, Cat#MT10017CV) culture medium supplemented with 10% fetal bovine serum (Gemini Bio-Products, Cat#100106) was 25 µL. On the day of assay, test drugs were diluted to 1 mM concentration in complete medium. 25 µL of this mixture was added to the cells already containing 25 µL medium to achieve a concentration of 500 µM. All treatments were done in triplicates. 25 µL of medium was removed from the first wells and added to the next well. This type of serial dilution was done 12 times and treated cells were then incubated at 37°C in a humidified CO _2_ incubator for 1 hour. Final concentrations of 250, 125, 62.5, 31.25, 15.62, 7.81, 3.9, 1.9, 0.97, 0.48, 0.24 and 0.12 µM were achieved upon addition of 25 µL of infection mix containing Ebola-GFP virus, Bafilomycin at a final concentration of 10 nM was used as a positive control drug. Infections were done to achieve a MOI of 0.05 to 0.15. Infected cells were incubated for 24 hours. 24 hours post-infection cells were fixed by immersing the plates in formalin for 24 hours at 4°C. Fixed plates were decontaminated and brought out of the BSL-4. Formalin from fixed plates was decanted and plates were washed thrice with PBS. EBOV-infected cells were stained for nuclei using Hoechst at 1:50,000 dilution and plates were imaged. Nuclei (blue) and infected cells (green) were counted using CellProfiler software (Broad Institute)- Version 2.1.1. Total number of nuclei (blue) was used as a proxy for cell numbers and a loss of cell number was assumed to reflect cytotoxicity. Concentrations where total cell numbers were 20% less than the control were rejected from the analysis.

## Results

### Machine learning

**Figure 1.**
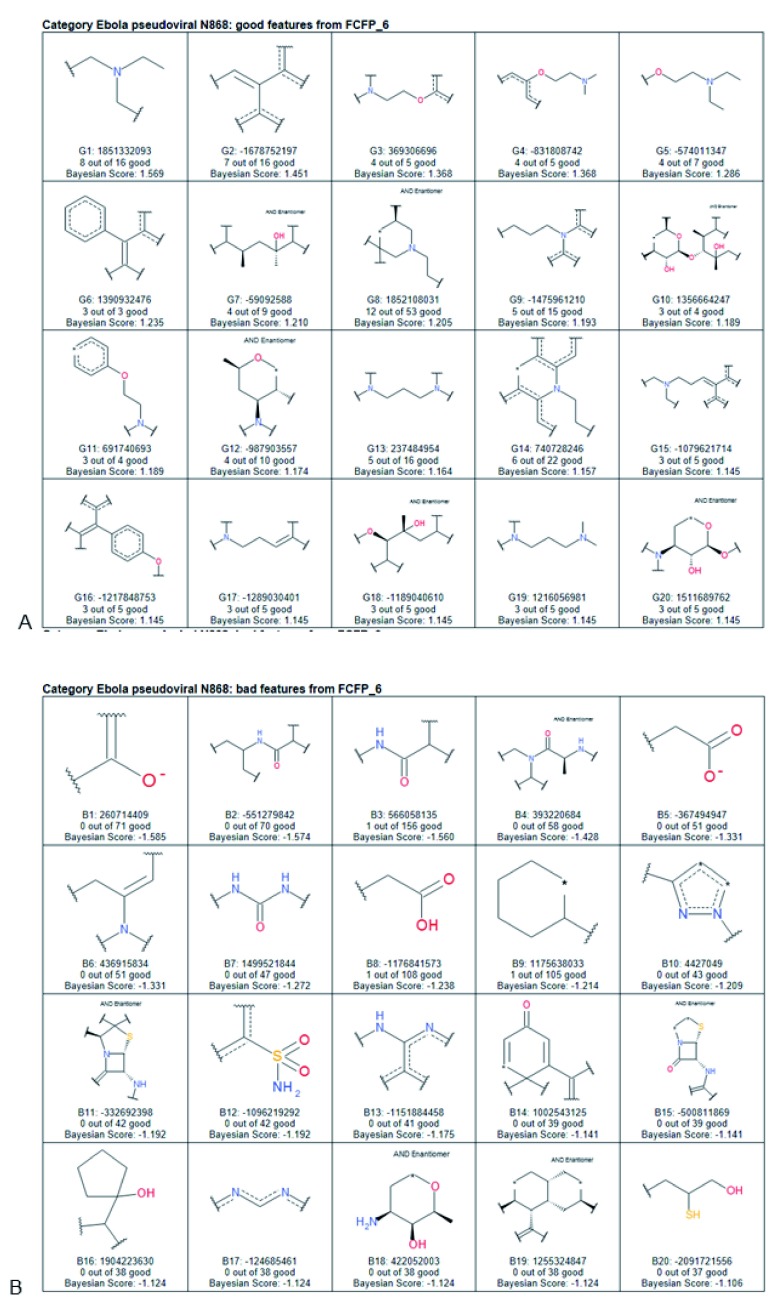
**A**. Active and **B**. Inactive features for the Discovery Studio pseudotype Bayesian model.

**Figure 2.**
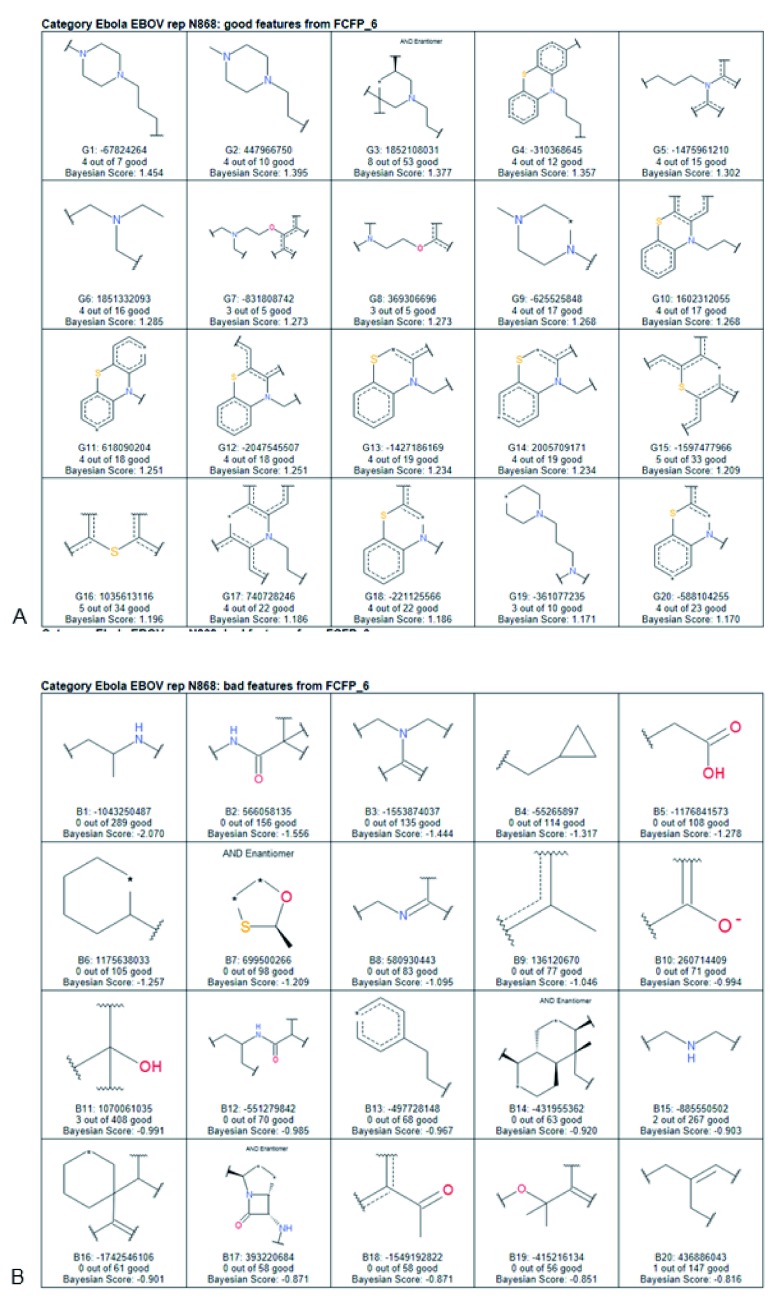
**A**. Active and **B**. Inactive features for the Discovery Studio EBOV replication model.

Using 5-fold cross validation the Bayesian approach ( Data S1 and Data S2) performed the best for the EBOV replication data and was equivalent for the RP Forest approach ( Table 1) and was better than SVM ( Data S3 and Data S4) for the pseudotype data. The Open Bayesian models had ROC scores slightly lower than the Bayesian models built with Discovery Studio. A more exhaustive cross validation for the Bayesian models is the ‘leave out 50% repeated randomly 100 times’ which produced ROC values greater than 0.8 and were comparable to the 5-fold cross validation data. This indicated the models are stable. For the EBOV pseudotype assay, alkoxyethylamino was a common feature amongst active compounds in the training set, as were 1,3-diaminopropyl and saturated six-member heterocycles with an oxygen and perhaps an additional heteroatom in the ring ( Figure 1A). Training set inactives commonly featured carboxylic acids, N,N'-disubstituted ureas, secondary and tertiary amides, pyrazoles, aromatic sulfonamides, tertiary cyclopentanols, 1,2-mercaptoethanol, and penams ( Figure 1B). For the replication assay training set, active features included piperazine, phenothiazine, tertiary amines, and alkoxyethylamino ( Figure 2A). Inactive features included secondary amides, disubstituted amines, cyclopropylmethyl, carboxylic acids, 1,3-oxathiolanes, tertiary alcohols, phenethyl, and penams ( Figure 2B). An actives feature common between both assays/models was alkoxyethylamino. Inactives features in common between both were carboxylic acids, secondary amides, penams and tertiary alcohols, which may relate to properties which prevent the molecules from accessing cellular sites of viral activity.

**Table 1.** Machine learning model cross validation Receiver Operator Curve (ROC) statistics.

Models (training set 868 compounds)	RP Forest (Out of bag ROC)	RP Single Tree (With 5 fold cross validation ROC)	SVM (with 5 fold cross validation ROC)	Bayesian (with 5 fold cross validation ROC)	Bayesian (leave out 50% × 100 ROC)	Open Bayesian (with 5 fold cross validation ROC)
Ebola replication (actives = 20)	0.70	0.78	0.73	0.86	0.86	0.82
Ebola Pseudotype (actives = 41)	0.85	0.81	0.76	0.85	0.82	0.82

**Figure 3.**
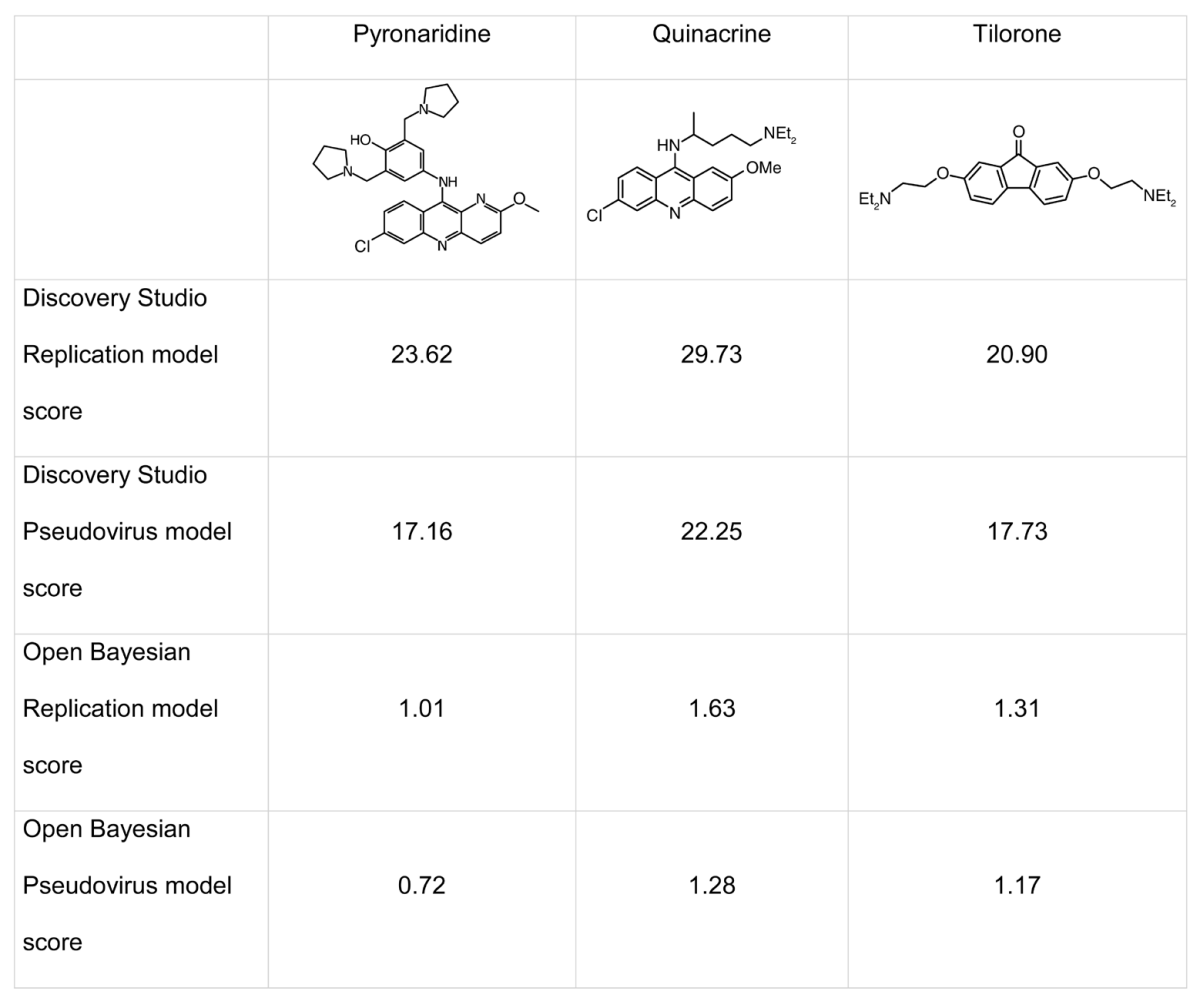
Molecules scoring well with the Ebola Bayesian models. For comparison, chloroquine scored 31.38 in the replication Discovery Studio Bayesian model, 24.55 in the Discovery Studio Pseudovirus Bayesian model, 1.63 in the Open Bayesian Replication model and 0.51 in the Open Bayesian Pseudovirus model.

The MicroSource Spectrum set of 2320 compounds was then scored with both Bayesian models ( Data S5). Predicted actives were quantified as to their chemical similarity, or distance, from molecules in the training set. When excluding compounds in the training set (as well as antipsychotics and other less desirable CNS active compounds), those scoring highly were considered most interesting and included the antiviral tilorone, the antimalarials quinacrine and pyronaridine ( Figure 3). Perhaps not surprisingly, tertiary amines scored particularly well. These molecules were also scored with the open Bayesian models ( Data S6) and all replication models scored the compounds highly (values close to or greater than 1). None of these three compounds has been described in recent reviews of small molecules with activity against EBOV ^14– 16^, to our knowledge.

### Pharmacophore

**Figure 4.**
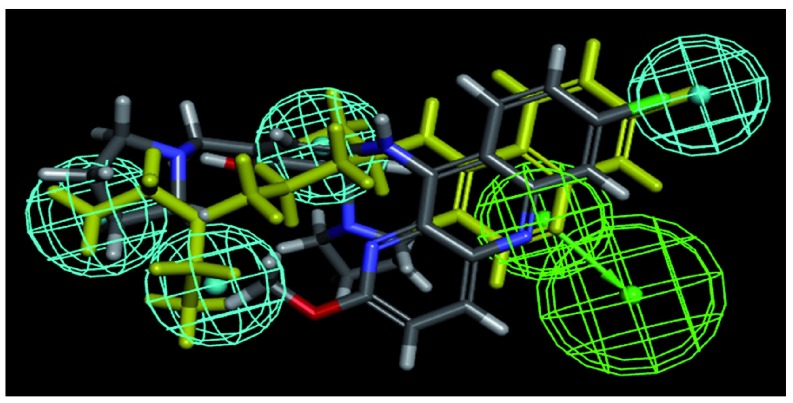
Pyronaridine mapped to a previously published pharmacophore based on compounds active against Ebola virus *in vitro*. Fit score of 3.60 (Chloroquine (yellow) = 4.21).

The MicroSource set had previously been screened with the published Ebola common feature pharmacophore ^26, 27^, using the van der Waals surface of amodiaquine (which was more potent than chloroquine ^3^) to limit the number of hits retrieved ^42– 44^. Two of the three selected – compounds quinacrine (fit score 2.59) and tilorone (fit score 3.65) – were retrieved previously. We therefore used the ligand pharmacophore mapping to map pyronaridine to the pharmacophore without the van der Waals surface ( Figure 4, Fit score of 3.60 suggested this was a good match to pharmacophore features).

### *In vitro* testing

**Figure 5.**
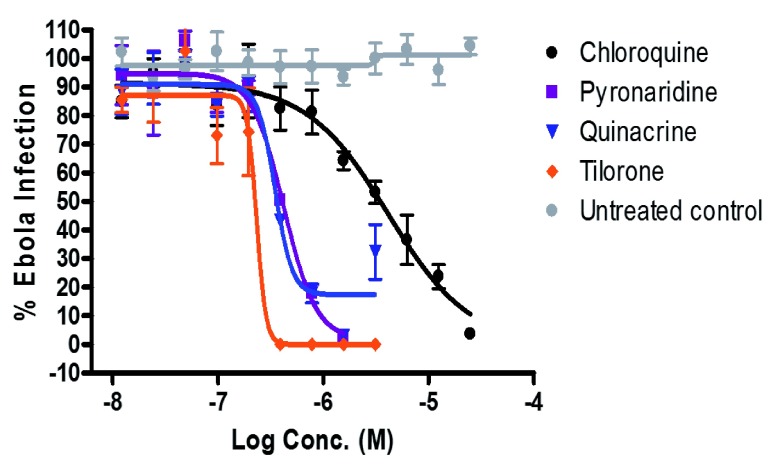
Effect of drug treatment on infection with Ebola-GFP. Cells were treated and then challenged with Ebola virus encoding GFP. Infection efficiency was calculated as infected cells (expressing GFP)/total cells and normalized to infection efficiency seen in the untreated control. Shown is one representative experiment where each point is the average of 3 independent measurements of infection +/- standard deviation. Dose response curves were fitted by non-linear regression.

The three selected compounds were tested *in vitro* alongside the positive control chloroquine which gave an expected dose response curve ( Figure 5, Table 2). Quinacrine, pyronaridine and tilorone, were tested *in vitro* and had EC _50_ values of 350, 420 and 230 nM, respectively which were lower than for chloroquine 4.0 μM. Several images created in this study illustrate the results of this high content screen ( Data S7).

**Table 2.** Effect of drug treatment on infection with Ebola-GFP (n=3 per compound). The cytotoxicity of compounds are represented as a 50% cytotoxicity concentration (CC _50_) estimated by the lowest concentration of drug that produced ≥ 50% loss in cell number by nuclei counting.

Compound	EC _50_ (μM) [95% CI]	Cytotoxicity CC _50_ (µM)
Chloroquine	4.0 [1.0–15]	250
Pyronaridine	0.42 [0.31–0.56]	3.1
Quinacrine	0.35 [0.28–0.44]	6.2
Tilorone	0.23 [0.09–0.62]	6.2

## Discussion

Our recent work on neglected diseases has shown that we can learn from existing assay datasets. Specifically we have previously analyzed large datasets for *Mycobacterium tuberculosis* to build machine learning models that use single point data, dose-response data ^43, 45^, combine bioactivity and cytotoxicity data (e.g. Vero, HepG2 or other model mammalian cells) ^28, 29, 46^ or combinations of these sets ^47, 48^. These models in turn have been validated with additional non-overlapping datasets, demonstrating that it is possible to use publically accessible data to find novel *in vitro* active antituberculars. We have also applied the same approach recently to identify a molecule with *in vitro* and *in vivo* activity against *T. cruzi* ^30^. In the current study we found that different machine learning methods produced similar 5-fold cross validation data, although the Bayesian models had ROC values consistently above 0.80, which is preferable. One of the issues with computational models is that they are rarely accessible to others due to the commercial software licensing requirements. We have previously showed that models built with open source tools can produce validation statistics comparable to commercial modeling tools ^49^. We recently made “function class fingerprints of maximum diameter 6” (FCFP6) and “extended connectivity (ECFP6) fingerprints,” open source and have described their implementation with the Chemistry Development Kit (CDK) ^50^ components ^41^. In addition we described an open source Bayesian algorithm that can be used with these descriptors ^39, 40^. One way to make such models more accessible is to use mobile devices for their delivery and we have developed cheminformatics mobile apps ^41, 51– 55^. Several of these apps combine Bayesian models and open source fingerprint descriptors to enable models that can be used within a mobile app (TB Mobile, MMDS, Approved Drugs and MolPrime). This enables a scientist to select a molecule and score it with models. In the current study we used the same training sets for the anti-EBOV activity using replication and pseudotype screening data to build open source models that we can share with the community ( http://molsync.com/ebola/).

The Bayesian models allowed us to select three compounds from the MicroSource compound library that scored highly and were not in the model training sets. The Open Bayesian models also scored the three hits favorably, which bodes well for screening other compounds of interest. Two of these molecules had also been identified with our earlier pharmacophore model which may be indicative of binding to VP35 ^26^. When tested *in vitro* the three compounds possessed EC _50_ values 230–420 nM, much lower than the positive control chloroquine (EC _50_ 4.0 μM) used in this study and identified previously ^3^. Tilorone is an investigational agent that has been known for over 40 years as an antiviral ^56^ and is an inducer of interferon in mice ^57^. It has been shown to possess a broad array of biological activities including cell growth inhibition in PC3 CDK5dn prostate cancer cells (IC _50_ 8–12 μM) ^58^, inhibition of Primase DnaG from *Bacillus anthracis* (IC _50_ 7.1 μM) ^59^, in a mouse model of pulmonary fibrosis it decreased lung hydroxyproline content and the expression of collagen genes ^60^, α7 nicotinic receptor (nAChR) agonist activity (K _i_ 56 nM) ^61^, activated human alpha7 nAChR with an EC _50_ value of 2.5 μM ^62^, radioprotective activity ^63^, potent modulation of HIF-mediated gene expression in neurons with neuroprotective properties ^64^ and induction of the accumulation of glycosaminoglycans, delay infectious prion clearance, and prolong prion disease incubation time ^65^. Quinacrine is an old antimalarial drug now more widely used as an antiprotozoal for the treatment of giardiasis ^66^ and as an anthelmintic. Pyronaridine is a potent antimalarial (IC _50_ 13.5 nM) ^67^, has activity against *Babesi*a spp. ^68^, is active *in vitro* (EC _50_ 225 nM) and *in vivo* (85.2% efficacy 4 days treatment at 50 mg/kg) against *T. cruzi* ^30^ and is a P-glycoprotein inhibitor ^69^. Pyronaridine is used in combination with artesunate in the European Medicines Agency approved Pyramax ^70^ which has performed well in clinical trials for malaria ^71^. As this molecule has already been approved this may have a more direct path to clinical testing if it is found to be active in standard animal models infected with the Ebola Virus.

As stated before in perspectives by us ^72^ and others ^2, 16, 20, 73^, the fact that approved drugs may be repurposed for other diseases should not be viewed as a negative aspect of the small molecules, belying undesirable target promiscuity ^74^. Instead, we prefer to reference recently published crystallographic analyses ^75^ demonstrating that small molecules may bind multiple proteins in different types of binding sites and with distinct conformations to ultimately facilitate molecular repurposing. While it would be most desirable to repurpose an approved drug and, thus, catapult a discovery effort into a Phase II trial, one should not ignore the significance of utilizing the discovery of a new use for an old drug to seed efforts in the lead optimization phase ^76^. Such an expedited program would be expected to have a high probability of producing novel small molecules, closely related to or inspired by the drug, with the opportunity to translate quickly to clinical trials.

In summary, this study has added to the previous work that identified several FDA approved compounds active against EBOV *in vitro*. Future work may include identification of targets using computational or experimental approaches. We propose that these three molecules may warrant further evaluation *in vivo* as they are significantly more active than chloroquine. Larger scale virtual screening could be performed on the millions of commercially available molecules or more complete sets of approved and older no longer used drugs than have already been screened. These computational efforts can then prioritize molecules for testing. Such an approach may be a useful way to leverage the HTS data that has already been developed at great cost. In this study we have focused on just the data from a single group ^3, 31^ but it may also be possible to combine this with the data from the other high throughput screens ^2, 16, 17^ to provide a much larger training set. There is also the opportunity to apply many different computational approaches beyond those described here to identify whole cell active compounds against EBOV. Ultimately, we should be able to identify additional compounds that could be immediately useful to treat patients with the disease while we await the approval of a vaccine.

## Data availability

The data referenced by this article are under copyright with the following copyright statement: Copyright: © 2017 Ekins S et al.

Data associated with the article are available under the terms of the Creative Commons Zero "No rights reserved" data waiver (CC0 1.0 Public domain dedication). 

Supplemental data contains results from Bayesian models and SVM models as well as the output of predictions with Bayesian models and open Bayesian models.

The training sets used in the models are available as SDF files ( http://molsync.com/ebola/).

## Supplementary materials

**Supplemental data S1–S4 and S6, S7.**

Supplemental data S1. Pseudotype Bayesian model, Supplemental data S2. EBOV replication Bayesian, Supplemental Data 3. SVM output file for Pseudotype model, Supplemental Data S4. SVM output file for EBOV replication model, Supplemental Data S6. Predictions for Ebola activity using Open Bayesian models in the MMDS app, Supplemental Data S7. High content screening images illustrating inhibition of Ebola and cytotoxic concentration.

http://dx.doi.org/10.5256/f1000research.7217.s110159

**Supplemental data S5.**

MicroSource predictions with Bayesian models xl file.
