# Supplementary material for: Machine learning models identify molecules active against the Ebola virus in vitro
Source: F1000Res. 2017 Jan 17;4:1091. Originally published 2015 Oct 20. [Version 3] doi: 10.12688/f1000research.7217.3 (PMC4706063; doi:10.12688/f1000research.7217.3)
Supplement: Supplementary file 1 [file f1000research-4-11510-s0001.tgz › 0c7d693a-50d0-41bf-9be0-c91db97ff2d3.docx]

Supplemental data

## Article

## Machine Learning Models Identify Molecules Active Against the Ebola Virus *In Vitro*

## Sean Ekins^1,2,3*^, Joel S. Freundlich^4^, Alex M. Clark^5^, Manu Anantpadma^6^, Robert A. Davey^6^ and Peter B. Madrid^7^

## ^1^ Collaborations in Chemistry, 5616 Hilltop Needmore Road, Fuquay-Varina, NC 27526, USA.

## ^2^ Collaborations Pharmaceuticals Inc, 5616 Hilltop Needmore Road, Fuquay-Varina, NC 27526, USA.

## ^3^Collaborative Drug Discovery, 1633 Bayshore Highway, Suite 342, Burlingame, CA 94010, USA

## ^4^ Departments of Pharmacology & Physiology and Medicine, Center for Emerging and Reemerging Pathogens, UMDNJ – New Jersey Medical School, 185 South Orange Avenue Newark, NJ 07103, USA.

## ^5^ Molecular Materials Informatics, Inc., 1900 St. Jacques #302, Montreal H3J 2S1, Quebec, Canada

## ^6^ Texas Biomedical Research Institute, San Antonio, TX 78227, USA.

## ^7^ SRI International, 333 Ravenswood Avenue, Menlo Park, CA 94025, USA.

* To whom correspondence should be addressed. Sean Ekins, E-mail address: ekinssean@yahoo.com, Phone: +1 215-687-1320 Twitter: @collabchem

**Supplemental data S1. Pseudotype bayesian model**

| \| ROC score is 0.847 (leave-one-out). Best cutoff for this model is 0.812. \| \| --- \| |
| --- | --- |
| \| \| \| **5-Fold Cross-Validation Result** \| \| \| \| \| \| \| \| \| \| \| --- \| --- \| --- \| --- \| --- \| --- \| --- \| --- \| --- \| --- \| \| **Model Name** \| **ROC Score** \| **ROC Rating** \| **True Positive** \| **False Negative** \| **False Positive** \| **True Negative** \| **Sensitivity** \| **Specificity** \| **Concordance** \| \| Ebola pseudoviral N868 \| 0.846 \| Good \| 39 \| 2 \| 176 \| 651 \| 0.951 \| 0.787 \| 0.795 \| \| \| --- \| --- \| --- \| --- \| --- \| --- \| --- \| --- \| --- \| --- \| --- \| --- \| --- \| --- \| --- \| --- \| --- \| --- \| --- \| --- \| --- \| --- \| --- \| --- \| --- \| --- \| --- \| --- \| --- \| --- \| --- \| \| 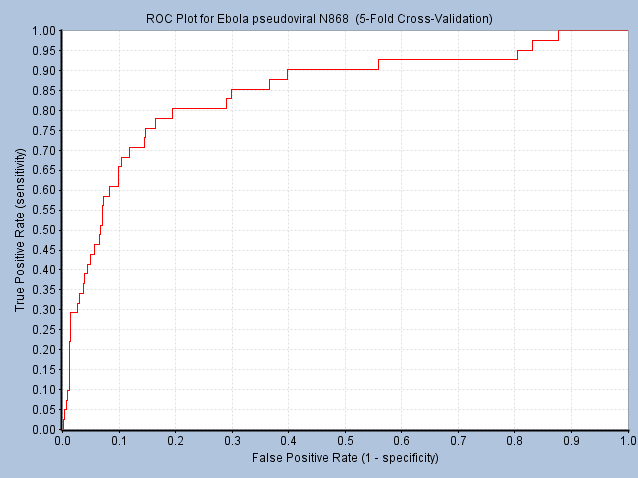  Leave out 50% x 100 fold cross validation   \| External_ROC_Score \| Internal_ROC_Score \| Concordance \| Specificity \| Sensitivity \| \| --- \| --- \| --- \| --- \| --- \| \| 0.82 \| 0.82 \| 79.98 \| 80.52 \| 68.90 \| \| 0.05 \| 0.04 \| 7.60 \| 8.39 \| 12.40 \| \| \| \| --- \| --- \| --- \| --- \| --- \| --- \| --- \| --- \| --- \| --- \| --- \| --- \| --- \| --- \| --- \| --- \| --- \| --- \| --- \| --- \| --- \| --- \| --- \| --- \| --- \| --- \| --- \| --- \| --- \| --- \| --- \| --- \| --- \| --- \| --- \| --- \| --- \| --- \| --- \| --- \| --- \| --- \| --- \| --- \| --- \| --- \| --- \| --- \| |

**Supplemental data S2. EBOV replication Bayesian**

| \| ROC score is 0.858 (leave-one-out). Best cutoff for this model is 6.770. See ModelDescription.html for more detailed information about this model. \| \| --- \| |
| --- | --- |
| \| \| \| **5-Fold Cross-Validation Result** \| \| \| \| \| \| \| \| \| \| \| --- \| --- \| --- \| --- \| --- \| --- \| --- \| --- \| --- \| --- \| \| **Model Name** \| **ROC Score** \| **ROC Rating** \| **True Positive** \| **False Negative** \| **False Positive** \| **True Negative** \| **Sensitivity** \| **Specificity** \| **Concordance** \| \| Ebola EBOV rep N868 USES CHLOROQUINE AND TOREMIFENE \| 0.867 \| Good \| 19 \| 1 \| 239 \| 609 \| 0.950 \| 0.718 \| 0.724 \| \| \| --- \| --- \| --- \| --- \| --- \| --- \| --- \| --- \| --- \| --- \| --- \| --- \| --- \| --- \| --- \| --- \| --- \| --- \| --- \| --- \| --- \| --- \| --- \| --- \| --- \| --- \| --- \| --- \| --- \| --- \| --- \| \| 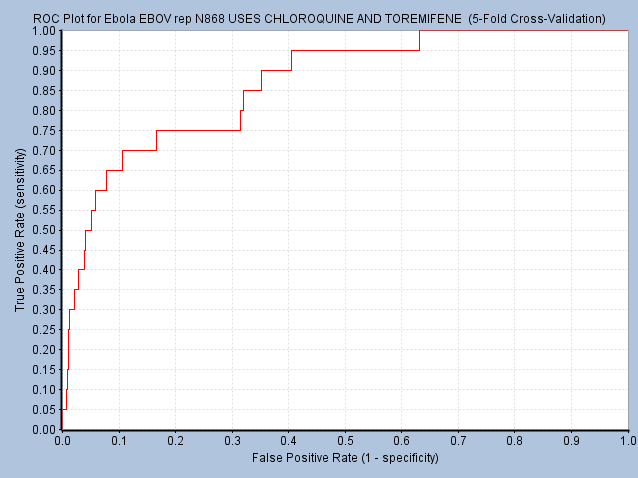 \| \| \| --- \| --- \| --- \| --- \| --- \| --- \| --- \| --- \| --- \| --- \| --- \| --- \| --- \| --- \| --- \| --- \| --- \| --- \| --- \| --- \| --- \| --- \| --- \| --- \| --- \| --- \| --- \| --- \| --- \| --- \| --- \| --- \| --- \| |

Leave out 50% x 100 fold cross validation

| External_ROC_Score | Internal_ROC_Score | Concordance | Specificity | Sensitivity |
| --- | --- | --- | --- | --- |
| 0.84 | 0.85 | 75.66 | 75.81 | 67.67 |
| 0.05 | 0.05 | 13.57 | 14.26 | 21.07 |

**Supplemental Data S3. SVM output file for Pseudotype model**

FitSummary

Call:

svm(formula = form, data = xy, type = type, kernel = tolower("Radial"),

gamma = gamma, cost = cost, probability = prob, fitted = TRUE,

epsilon = epsilon, nu = nu, coef0 = coef0, degree = degree, scale = TRUE)

Parameters:

SVM-Type: C-classification

SVM-Kernel: radial

cost: 2

gamma: 0.007352941

Number of Support Vectors: 307

( 266 41 )

Number of Classes: 2

Levels:

0 1

Cross-validation results (5-fold):

Gamma Cost ROC Score Best

1 0.007353 1 0.7538

2 0.007353 2 0.7598 ***

Contingency Table (best CV model):

Predicted

Actual 0 1

0 823 4

1 41 0

All-data model results (non-cross-validated):

Settings used:

Gamma Cost

0.007352941 2

ROC Score: 0.9997

Contingency Table (all-data model):

Predicted

Actual 0 1

0 827 0

1 13 28

FitPlot

Binary Property

**Supplemental Data S4. SVM output file for EBOV replication model**

FitSummary

Call:

svm(formula = form, data = xy, type = type, kernel = tolower("Radial"),

gamma = gamma, cost = cost, probability = prob, fitted = TRUE,

epsilon = epsilon, nu = nu, coef0 = coef0, degree = degree, scale = TRUE)

Parameters:

SVM-Type: C-classification

SVM-Kernel: radial

cost: 2

gamma: 0.007352941

Number of Support Vectors: 222

( 202 20 )

Number of Classes: 2

Levels:

0 1

Cross-validation results (5-fold):

Gamma Cost ROC Score Best

1 0.007353 1 0.7235

2 0.007353 2 0.7263 ***

Contingency Table (best CV model):

Predicted

Actual 0 1

0 845 3

1 20 0

All-data model results (non-cross-validated):

Settings used:

Gamma Cost

0.007352941 2

ROC Score: 1

Contingency Table (all-data model):

Predicted

Actual 0 1

0 848 0

1 5 15

FitPlot

Binary Property

**Supplemental Data S6. Predictions for Ebola activity using Open Bayesian models in the MMDS app.** Higher scores are more likely to be active.

**
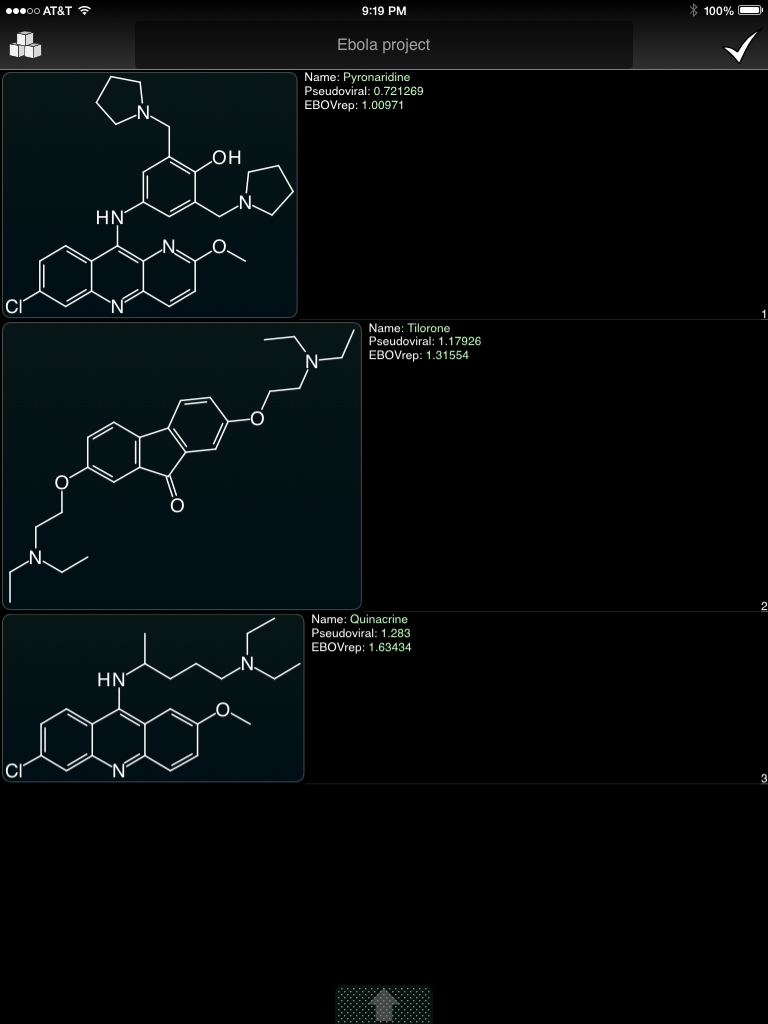
**

**Supplemental Data S7. High content screening images illustrating inhibition of Ebola and cytotoxic concentration.**

**
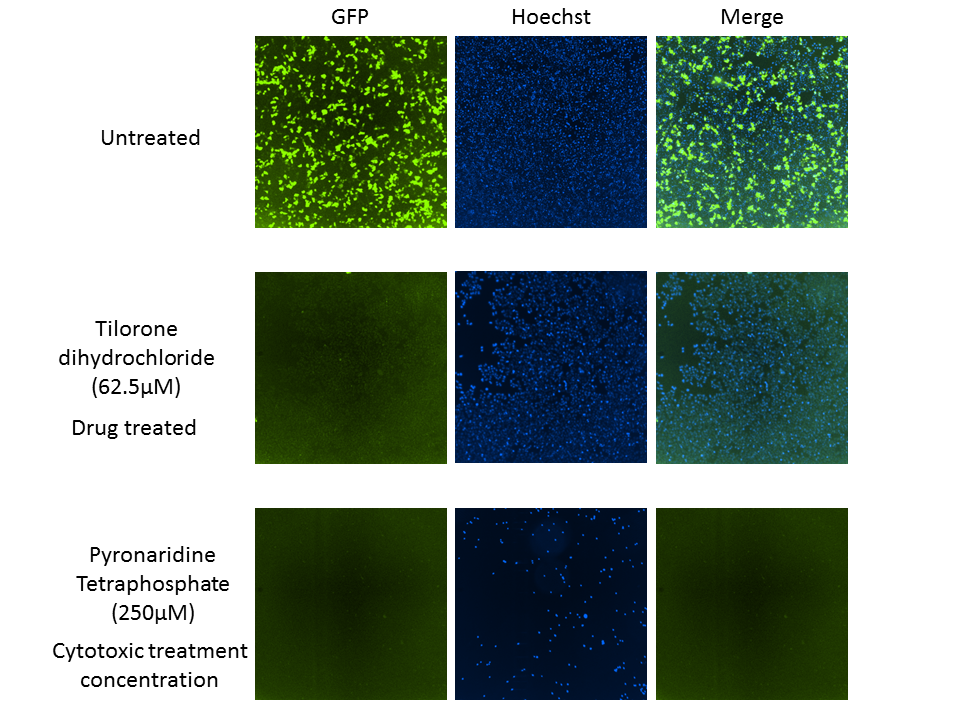
**
