# Supplementary material for: Machine learning models identify molecules active against the Ebola virus in vitro
Source: F1000Res. 2017 Jan 17;4:1091. Originally published 2015 Oct 20. [Version 3] doi: 10.12688/f1000research.7217.3 (PMC4706063; doi:10.12688/f1000research.7217.3)
Supplement: Peer review history

# Peer review: Referee response for version 3

**DOI:** 10.5256/f1000research.11510.r19459

**Published:** 2017-01-18

**Authors:** Sanja Glisic

**Affiliations:** 1 Center for Multidisciplinary Research, Vinča Institute of Nuclear Sciences, University of Belgrade, Belgrade, Serbia

I have read this submission. I believe that I have an appropriate level of expertise to confirm that it is of an acceptable scientific standard.

# Peer review: Referee response for version 1

**DOI:** 10.5256/f1000research.7773.r11173

**Published:** 2015-12-03

**Authors:** Sandeep Chakraborty

**Affiliations:** 1 Plant Sciences Department, University of California, Davis, CA, USA

Ekins *et al. *have presented a crisp and lucid manuscript on a very relevant topic. They have presented a methodology that implements machine learning techniques to learn from known active and inactive compounds (an ever increasing set, that will tend to provide improved results as time goes by), and score a larger set of compounds (MicroSource Spectrum set of 2320 compounds). The *in silico *methodology described here provides an excellent method to quickly screen known compounds for possible therapies (against Ebola in particular), and other viruses in general. Finally, they demonstrate ( *in vitro*) the increased effectiveness of three compounds - the antiviral tilorone and the antimalarials quinacrine and pyronaridine - in comparison to the known active chloroquine (albeit at a higher cytotoxicity) in inhibiting viral infection of HeLa cells. Further, their efforts in ensuring open-access to such tools is commendable as the next pathogen caused humanitarian crisis looms in some nations.

The biggest open question is how good are the molecular descriptors, and how much of this is serendipitous. For example, I find it hard to believe that molecular weight and the number of rotatable bonds can be good predictors of drug-protein interactions (although I may be wrong). I have been investigating promiscuous ligand protein interactions for a couple of years on a molecular basis ( http://journals.plos.org/plosone/article?id=10.1371/journal.pone.0032011, http://f1000research.com/articles/2-286/v3). One interesting example (unpublished) is suramin used in the treatment of African sleeping sickness (African trypanosomiasis) and river blindness (onchocerciasis), infections caused by parasites. Suramin binds eight non-homologous proteins in the PDB database, through different parts of the molecule and in binding sites that share little similarity in residues involved. Also, the molecule (in addition to the protein) undergoes conformational changes, underscoring the difficulty of computational methods to model such interactions. In the face of such data, the m/c learning models appear too simplistic.

Also, it is not completely clear why the 23, 31 and 34th compound was chosen from the Table S5, which is ordered on column H (all three have amines, don't the previous ones have it?).

Some minor comments:

1. It would be an interesting case study to evaluate how favipiravir (which I understand is yet to be clinically approved in the US, but approved in Japan) and BCX4430 would rank through the m/c learning methodology.
2. It would be good to have a set of images, and the corresponding nuclei counts obtained from CellProfiler. Is there a way to quantify the color green as a measure of viral infection?
3. AlogP as a molecular descriptor has not been explained (page 3).

I have read this submission. I believe that I have an appropriate level of expertise to confirm that it is of an acceptable scientific standard.

# Response

**Published:** 2015-12-19

**Authors:** Sean Ekins

**Affiliations:** Collaborations in Chemistry, USA

*We thank the reviewer for their constructive feedback.*

The approach we have taken uses FCFP_6 fingerprints as well as 8 interpretable descriptors, and therefore the models do not depend on molecular weight and rotatable bond number. On the whole this approach has been remarkable useful for predicting whole cell activity as we described for mycobacterium tuberculosis, T. Cruzi and now Ebola. In all cases we are not considering a single target. This suggests the machine learning approach and descriptors used can discern active molecule features from those that are inactive and identify new molecules.

The three compounds were chosen as those above them in the list were either compounds in the training set or antipsychotics and other CNS acting compounds etc. which were deemed less desirable.

The model could certainly be used to predict additional molecules. These two suggested by the reviewer are structurally distinct from any of the actives in our current training set. We didn’t identify any of the classical antiviral polymerase-looking compounds in our screening against Ebola. However we have previously collated and described many other diverse compounds active against Ebola *in vitro* as described in this manuscript. Perhaps the next step would be to utilize all of the different HTS screening data to build a combined model that considers this structural diversity and may overcome limitations in the current models.

Response: we have now added a new figure with cell images (S7).

Response: AlogP is a widely used measure of hydrophobicity and we have refered to its use along with the other descriptors in the previous machine learning papers.

# Peer review: Referee response for version 1

**DOI:** 10.5256/f1000research.7773.r10995

**Published:** 2015-11-10

**Authors:** Sanja Glisic

**Affiliations:** 1 Center for Multidisciplinary Research, Vinča Institute of Nuclear Sciences, University of Belgrade, Belgrade, Serbia

Ekins and his colleagues by using machine learning models and molecular modeling have successfully identified from collection of 2320 compounds 3 promising anti Ebola compounds with *in vitro* nanomolar activity. It is a perfect example which confirms suitability of *in silico* approaches in selection of molecules against Ebola virus.

This result will be strengthened with suggestion of possible therapeutic target(s) of selected

candidate drugs by using resources of curated chemistry-to-protein relationships. Such information could help in further improvement of proposed therapeutic molecules, as well as for selection of some other candidates for Ebola drugs.

I have read this submission. I believe that I have an appropriate level of expertise to confirm that it is of an acceptable scientific standard.

# Response

**Published:** 2015-12-19

**Authors:** Sean Ekins

**Affiliations:** Collaborations in Chemistry, USA

*We thank the reviewer for their constructive feedback*

Response: We are unsure which resources the reviewer is referring to and how these would help identify the antiviral target. We have tried to extensively describe the known activities of the three compounds against various targets outside of viruses. In addition we have previously suggested such antimalarials with Ebola activity may dock into VP35. Preliminary docking results suggest pyronaridine may dock into the same site which is also indicated by the pharmacophore provided already (fig 4). We have added the statement “Future work may include identification of targets using computational or experimental approaches.” to the discussion.
